# Fabrication and evaluation of slow-release lignin-based avermectin nano-delivery system with UV-shielding property

**DOI:** 10.1038/s41598-021-02664-7

**Published:** 2021-12-01

**Authors:** Dongmei Mo, Xiangying Li, Yong Chen, Yang Jiang, Chunfang Gan, Yuanfei Zhang, Weiguo Li, Yanmin Huang, Jianguo Cui

**Affiliations:** 1 Guangxi Key Laboratory of Natural Polymer Chemistry and Physics, College of Chemistry and Material Science, Nanning Normal University, Nanning, 530001 People’s Republic of China; 2 Guangxi Tianyuan Biochemical Co. Ltd., Nanning, 530001 People’s Republic of China

**Keywords:** Field trials, Materials science, Process chemistry

## Abstract

Nanopesticide is one of the best pesticide formulation technologies to overcome the disadvantages of traditional pesticides, which has received great attention from the international community. Using high-speed emulsification and ultrasonic dispersion technology, an avermectin nano-delivery system (Av-NDs) with a particle size of 80–150 nm was prepared through embedding the pesticide molecule utilizing the cross-linking reaction between sodium lignosulfonate and p-phenylenediamine diazonium salt. The formulation and composition of Av-NDs were optimized, the morphology of Av-NDs was analyzed by scanning electron microscope, transmission electron microscope and dynamic light scattering, and the structure of Av-NDs was characterized by UV, IR and ^1^H NMR. Anti-photolysis and controlled-release tests show that the stability of Av-NDs is 3–4 times of the original avermectin (Av) and possesses the pH-responsive controlled release property. Furthermore, the insecticidal activity of Av-NDs is better than that of avermectin suspension concentrate (Av-SC). The Av-NDs with anti-photolysis and controlled-release characteristics is suitable for large-scale industrial production and is capable to be utilized as effective insecticide in the field.

## Introduction

In agricultural production, the use of pesticides plays an important role in ensuring stable and high yields of grain^1,2^. Due to various disadvantages of traditional pesticide formulations, such as poor water solubility, easy photolysis, and easy drift^3,4^, new pesticide formulations are constantly emerging. Nanopesticide is one of the best pesticide formulation technologies featuring good wettability and droplet extensibility, strong ability to absorb targets, UV resistance, oxidation resistance and increased bioavailability. Its sustained-release and targeted drug delivery can drastically improve the utilization rate of pesticides thus reduces the dosage of pesticides and the use of organic solvent^5–7^, and therefore overcomes the disadvantages of traditional pesticides^8–10^. Hence, extensive attention has been paid to the development of novel nanopesticide by the international community^11^.

As a renewable resource, lignin is widely distributed in almost all plants^12,13^. Lignin contains many different reactive groups, such as phenolic hydroxyl, carbonyl, conjugated double bond and sulfonic group^14,15^. According to different sources of lignin, they show different antioxidant activities^16,17^, anti-ultraviolet decomposition^18,19^ and biodegradation^20,21^. At present, lignin has been widely used as a matrix in the preparation of pesticide formulations^22–24^.

**Figure 1:**
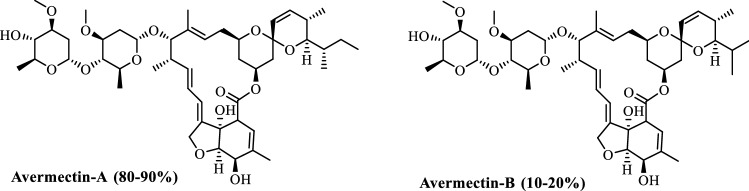
Chemical structure of avermectin.

Avermectin (composed of avermectin-A and avermectin-B, Fig. 1) is a sixteen-membered macrolide compound obtained from *Streptomyces avermitilis* in *Streptomyces*, and is widely used as insecticide, acaricide, nematicide for agricultural and veterinary with high efficiency, low toxicity and high selective insecticidal activity^25,26^. However, the avermectin bearing conjugated double bonds is environmental sensitive, easy to be photolyzed, and short of duration of drug efficacy^27^. In order to reduce its environmental sensitivity, and avoid the rapid loss of its insecticidal activity, the addition of stabilizers to avermectin formulations is often needed, alternatively, adsorption or wrapping of avermectin on certain substrates to prevent its degradation were also viable^28–32^. In addition, by using the lignin as a matrix material, avermectin nanoformulations were found to prolong the insecticidal effect of avermectin as well. For example, Li Y. et al. utilized the self-assembly of sodium lignosulfonate with CTAB (cetyltrimethylammonium bromide) to form a shell to wrap avermectin^33^. Subsequently, they converted alkaline lignin into a structure containing a tetravalent ammonium salt group, and then combined with SDBS (sodium dodecyl benzenesulfonate), an anionic surfactant, to form avermectin encapsulated matrix^34^. The interfacial polymerization of lignin and MDI (diphenyl methane diisocyanate) was used to generate lignin-polyurea microcapsules to encapsulate avermectin^35^. Deng, Y. et al. prepared a hollow sphere to encapsulate avermectin by the reaction of alkaline lignin with the diazonium salt of aniline^28^. The above avermectin nanoformulations obtained by lignin showed better anti-photolysis and slow-release effects.

Because abundant active diphenol groups occur, lignin can be cross-linked through the formation of azobenzene under the action of p-phenylenediamine diazonium salt to achieve the purpose of pesticide encapsulation. At the same time, azobenzene also has antioxidant properties. In this paper, we report a new method for preparing the avermectin nanoformulation with anti-photolysis and sustained-release properties by using p-phenylenediamine diazonium salt as a cross-linking agent for lignin and encapsulating avermectin.

## Materials and methods

### Materials

Avermectin (Av, Technical grade), Sodium lignosulfonate (Analytical Reagent (AR)), BY-125 (Castor oil polyoxyethylene ether, Technical grade), Ethylan NS-500LQ (Technical grade, Akzo Nobel), s-butyl acetate (AR), petroleum naphtha-100 (s-100, Technical grade), petroleum naphtha-200 (s-200, Technical grade), methyl oleate (Technical grade), Tween 60 (Technical grade), Tween 80 (Technical grade), Span 80 (Technical grade), Span 60 (Food grade), Span 40 (Food grade), Span 20 (Food grade), Distarch phosphate (Food grade), Acetylated distarch adipate (Food grade), Acetylated distarch phosphate (Food grade), Oxidized starch (Food grade), EL (polyoxyethylated castor oil, Technical grade), o-xylene (AR).

The commercial avermectin emulsifiable concentrates (Av-EC, 2.5%) and avermectin suspension concentrate (Av-SC, 2.5%) were provided from Guangxi Tianyuan Biochemical Co. Ltd., China. High performance liquid chromatography (HPLC) grade acetonitrile, methanol and ammonium hydroxide were purchased from Thermo Fisher Scientific Co., Ltd., China. Milli-Q water (18.2MΩ⋅cm, TOC ≤ 4 ppb) was used in all analytical experiments.

### Methods

#### Preparation of avermectin nano-delivery system

The Avermectin Nano-Delivery System (Av-NDs) was prepared via the cross-linking reaction between sodium lignosulfonate and p-phenylenediamine diazonium salt. First, 6.2 g of avermectin was dissolved in 30 mL of s-butyl acetate to obtain the avermectin solution **1**. Then, 4.0 g of sodium lignosulfonate was dissolved in 120 mL of deionized water by stirring. After the dissolution was completed, 3.8 g of emulsifier By-125 was added and continued stirring for 5 min until By-125 was fully dispersed. Next, the solution **1** was added to the sodium lignosulfonate solution under high-speed stirring at room temperature, and the stirring was continued for 10 min at a speed of 13,000 rpm (IKA T18 digital Ultra Turrax, IKA, Germany.) and cooled to 0–5 °C to obtain a coarse emulsion **2**. Meanwhile, 0.2 g of p-phenylenediamine was dissolved in 20 mL of 1 M H_2_SO_4_, cooled to 0–5 °C, 0.2 g of NaNO_2_ was added, and the mixture was stirred for 0.5 h to obtain solution **3**. Then, the solution** 3** was slowly added to the coarse emulsion **2** at 0–5 °C, and continued to stir at 600 rpm for 30 min. The resulting sample was then ultrasonically dispersed for 20 min under ice bath conditions (Ultrasonic Homogenizer JY 92-IIN, Ningbo Xinzhi Biotechnology Co., Ltd., Ningbo, China). Finally, 1.5 g of NS-500 LQ was add to the sample after sonication, and continued stirring at a speed of 800 rpm (Laboratory Disperser SDF 400, Foshan Yifu Machinery Co. Ltd., China) for 20 min to obtain the Av-NDs.

#### Characterization of Av-NDs

The hydrodynamic diameter (Dp) and polydispersity index (PDI) of Av-NDs were measured in triplicate by dynamic light scattering (DLS) using a Horiba SZ-100-Z Nano Particle Analyzer (HORIBA Instruments Co., Ltd, Japan) at 25 °C. The sample was diluted with deionized water to a solid content of about 0.1 wt%.

Identification of the chemical structure in Av-NDs was qualitatively determined via UV–Vis absorption spectrum (Shimadzu UV–Vis spectrophotometer, UV-2600, Shimadzu Instrument (Suzhou) Co. LTD) under the maximum absorption wavelength (λ_max_) of 245 nm, fourier transform infrared spectrum (Nicolet IS-10, Thermo Scientific; using the technical avermectin and the Av-NDs nanosphere powder in KBr) and ^1^H NMR (JNM-ECZ600R, JEOL Co. LTD, Japan; D_2_O was used as solvent).

The morphology of the nanospheres was verified by scanning electron microscope (SEM, Hitachi s-3400 N, Japan) operating at 15 kV using samples prepared by dropping on cleaned tinfoil, dried naturally. Morphology of the dried nanospheres was observed by transmission electron microscope (TEM, JEM-1200EX, JEOL Co. LTD, Japan) operating with 100 kV accelerating voltage, and the samples were prepared by mounting and drying the diluted Av-NDs on the carbon-coated copper grid at room temperature.

#### *Determination of Av content in Av-ND*_*S*_* by HPLC*

The Av content was determined by HPLC (Agilent 1260 LC, Agilent Technologies Co., Ltd., Germany) at 25 °C using a C18 analytical column (5 μm, 4.6 mm ∗ 150 mm, Aupos scientific, Germany) and 242 nm UV detector. The mobile phase was a mixture of methanol and water (82:18, v/v) and the flow rate was 1.00 mL/min.

#### Determination of avermectin loading in Av-NDs

The percentages of the avermectin loading capacity (LC) and encapsulation efficiency (EE) in Av-NDs were tested as follows: a certain amount of Av-NDs was centrifuged at 15,000 rpm for 10 min followed by three periods of washing-centrifuging, and the nanospheres were weighed after drying in a freeze dryer (SCIENTZ-12 N, Ningbo Xinzhi Biotechnology Co. Ltd, China). Then, the Av-NDs nanospheres and 20 mL of methanol were charged into a 25 mL volumetric flask, and the mixture was kept under the ultrasound for 1 h to make avermectin release completely. Subsequently, the solution was diluted to scale with methanol, and filtered with 0.22 µm microporous membrane. All were treated in triplicate. The content of avermectin was analyzed using UV–Vis spectrometer under a detection wavelength of 245 nm, and the LC and EE were calculated according to Eqs. (1) and (2), respectively.

1 $$ {\text{LC}}({\text{wt}}\% ) = \frac{{\text{m}}}{{\text{M}}} \times 100\% $$

2 $$ {\text{EE}}(\% ) = \frac{{\text{m}}}{{{\text{m}}_{{\text{o}}} }} \times 100\% $$

The m is the mass of avermectin loaded in Av-NDs, M is total mass of specimens, and m_o_ is the mass of avermectin added in specimens.

#### Controlled-Release behavior of Av-NDs

The release behavior of avermectin in the Av-NDs was determined by the dialysis method. A certain amount of Av-NDs was transferred to a dialysis bag (cutting Mw = 3500 Da). The bag was sealed and immersed into 100 mL of methanol/water mixture (7:3, v/v, pH = 7) in a wide-mouth bottle, and the bottle was placed in the shaking incubator at 25 °C with a shaking speed of 180 rpm. At predesigned time intervals, the solution was withdrawn from the bottle and an equal volume of fresh methanol/water mixture was supplemented into the bottle. The content of avermectin was analyzed by the UV–Vis spectrophotometer and the accumulated release (%) of avermectin was determined. Three replicates were performed at each interval in order to obtain the release curve of avermectin.

The releasing profile of Av-NDs in different pH (pH = 5.5, pH = 7.0 and pH = 9.0) was investigated using the same method as above, and the commercial Av-SC was used as a control. The pH value of the media was adjusted with a hydrochloric acid aqueous solution (0.1 mol/L) or sodium hydroxide aqueous solution (0.1 mol/L). The cumulative release rate of avermectin was calculated according to Eq. (3):

3 $$ {\text{Cumulative\;release\;rate}}:Q(\% ) = 100\% \times \frac{{\Sigma \left( {V \times Ct} \right)}}{W} $$

Q: cumulative release rate; W: total weight of avermectin in sample (mg); V: the volume of each sample taken (100 mL); Ct: the concentration of avermectin per sampling (mg/mL).

#### Photostability of avermectin in Av-NDs

The photostability of avermectin in Av-NDs was evaluated with the commercial Av-SC as a control. The sample was diluted with deionized water to the concentration (1 mg/mL) of avermectin and divided equally into 24-wells culture plates in 1 mL per well, and the culture plates were sealed with transparent plastic wrap. The sample was irradiated for a desired duration at 25 °C under an UV lamp (40 W) at 45 cm distance. At predesigned time intervals, the culture dish was taken out and the avermectin concentration of samples was determined using the UV–Vis absorption spectroscopy and the photolysis rate was calculated.

#### Storage stability of Av-NDs

The storage stability was examined as the following instructions. The Av-NDs was stored in a closed glass bottle at 4 °C and 54 °C for 14 days. Physical stability was assessed by the measurement of Dp and PDI using DLS. Chemical stability was evaluated by analyzing remaining avermectin in Av-NDs using HPLC.

#### Bioassay of Av-NDs

The toxicity test was conducted on *Mythimna separate walker*. The samples were diluted with deionized water to different concentrations (200, 40, 8, 1.6, and 0.32 ppm). Afterwards, the fresh maize leaves were cut into pieces of 6-8 cm long and then immersed in the above dispersions for 10 s and deionized water as a blank control. After air-drying, leaves were transferred to the 11 × 7.5 × 4 cm culture boxes with a filter paper at the bottom. Then, 40 of healthy third-instar *Mythimna separate* were gently introduced into each culture box with a brush and sealed with plastic cover possessing small holes. The larvae were housed in the pest control room under the conditions of light/dark = 16:8, temperature = 25 ± 2 °C and relative humidity = (75 ± 5)%, and considered dead if they could not be induced to move when touched with a brush. Mortality was assessed after treatment for 48 h, and four replications were carried out for comparison. The Av-SC was used as the positive controls. The mortality rate was calculated by Eq. (4).

4 $$ {\text{M}}({\text{mortality\;rate}},\% ) = \frac{{{\text{n}}/{\text{n}}_{{\text{o}}} - {\text{n}}\prime /{\text{n}}_{{\text{o}}} }}{{100 - {\text{n}}\prime /{\text{n}}_{{\text{o}}} }} \times 100\% $$

The n is the number of dead insects, n′ is the number of dead insects in the blank control and n_o_ is the total number of insects added to the box.

### Statistical analysis

All data of the experiment were expressed as mean standard deviation (S.D.) and significant difference between experimental replicates was examined using one-way ANOVA and Duncan’s multiple range test. The value of *p* < 0.05 indicates statistical significance.

## Results and discussion

### Fabrication of Av-NDs

**Figure 2:**
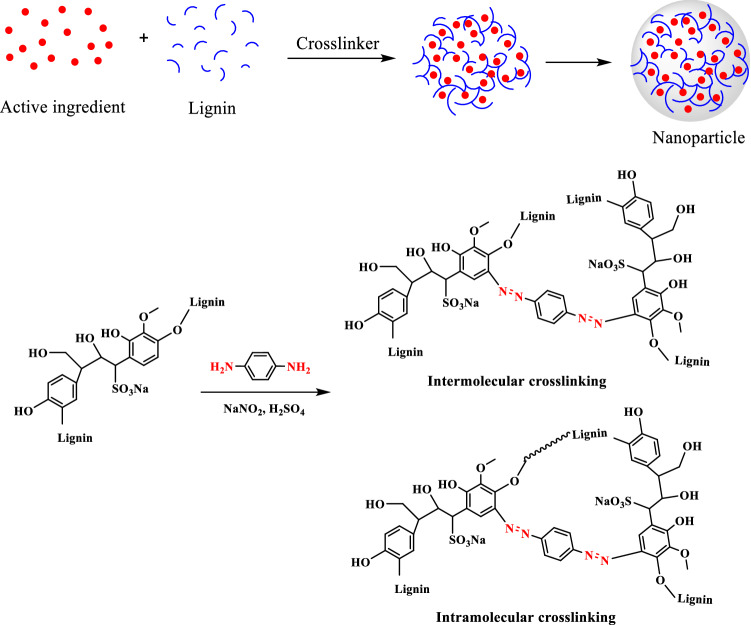
Fabrication mechanism of Av-NDs.

Using p-phenylenediamine diazonium salt as a cross-linking agent, avermectin was embedded in the nanospheres through the cross-linking reaction between sodium lignosulfonate and p-phenylenediamine diazonium salt, and then the nanoparticles formed were further dispersed and homogenized by ultrasonic dispersion method, and finally the Av-NDs was obtained. The preparation mechanism is shown in Fig. 2:

First, different solvents were used to dissolve avermectin, and the influences of the solvent on the particle size, PDI value and stability of the nanoformulation were investigated. The results showed that when s-butyl acetate was used as the solvent of avermectin, the nanoformulation obtained had smaller particle size and better storage stability. Furthermore, the effects of different types of emulsifiers and dispersants on the nanoformulation were studied (Supporting Materials), showing that when s-butyl acetate was used as the solvent, By-125 as the emulsifier and NS-500LQ as the dispersant, the resulting nanoformulation had the optimal mean particle size (121.4 ± 1.1 nm), size distribution (PDI: 0.244 ± 0.014), 72.07% LC and 72.63% EE.

### Microtopography analysis of Av-NDs

**Figure 3:**
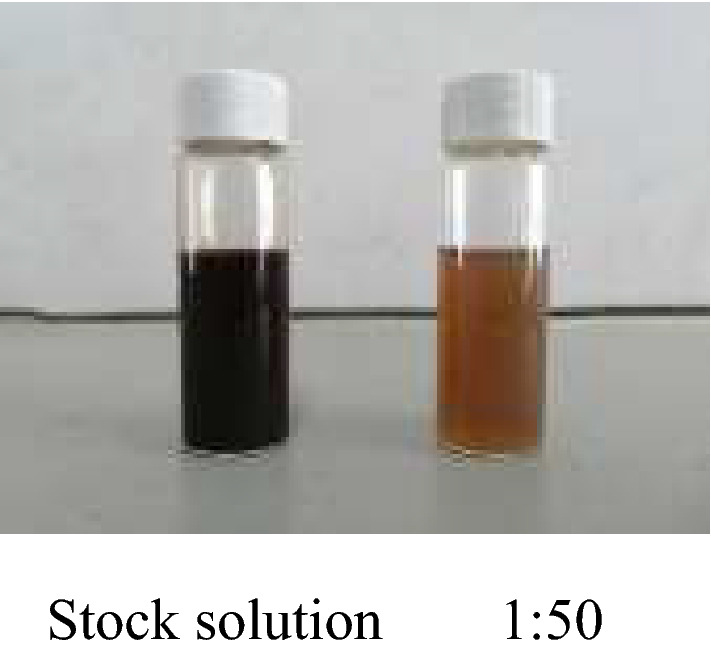
Appearance of Av-NDs.

Figure 3 shows the appearance of Av-NDs, which is a brown–black emulsion and no obvious change was observed after the cold and hot storage test. After the nanoformulation was diluted 30 times with distilled water, it became a translucent brown solution without any agglomeration.

**Figure 4:**
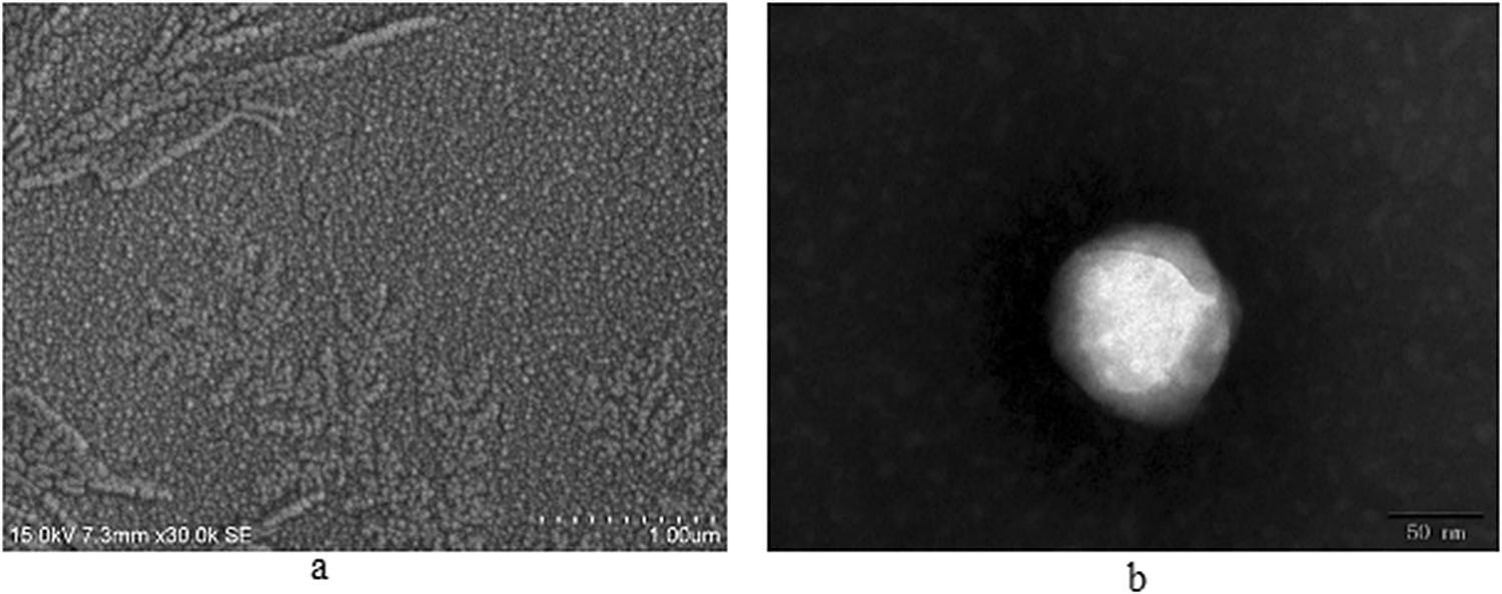
SEM images (**a**) and TEM images (**b**) of Av-NDs.

SEM and TEM images of the sample are shown in Fig. 4. As depicted in Fig. 4a, nanoparticles of Av-NDs are evenly distributed with clear contour, spherical in shape, and the particle sizes are located between the range of 80–150 nm, which are consistent with the result of measure by dynamic light scattering using a Horiba SZ-100-Z Nano Particle Analyzer (Mean size: 121.4 ± 1.1 nm; PDI: 0.244 ± 0.014). At the same time, there is no obvious agglomeration in the nanoparticles. In addition, it can be seen from the results of TEM (Fig. 4b) that the avermetin is irregularly dispersed inside the nanosphere, which is consistent with the entrapment nanoparticle structure.

### Structure characterization of Av-NDs

**Figure 5:**
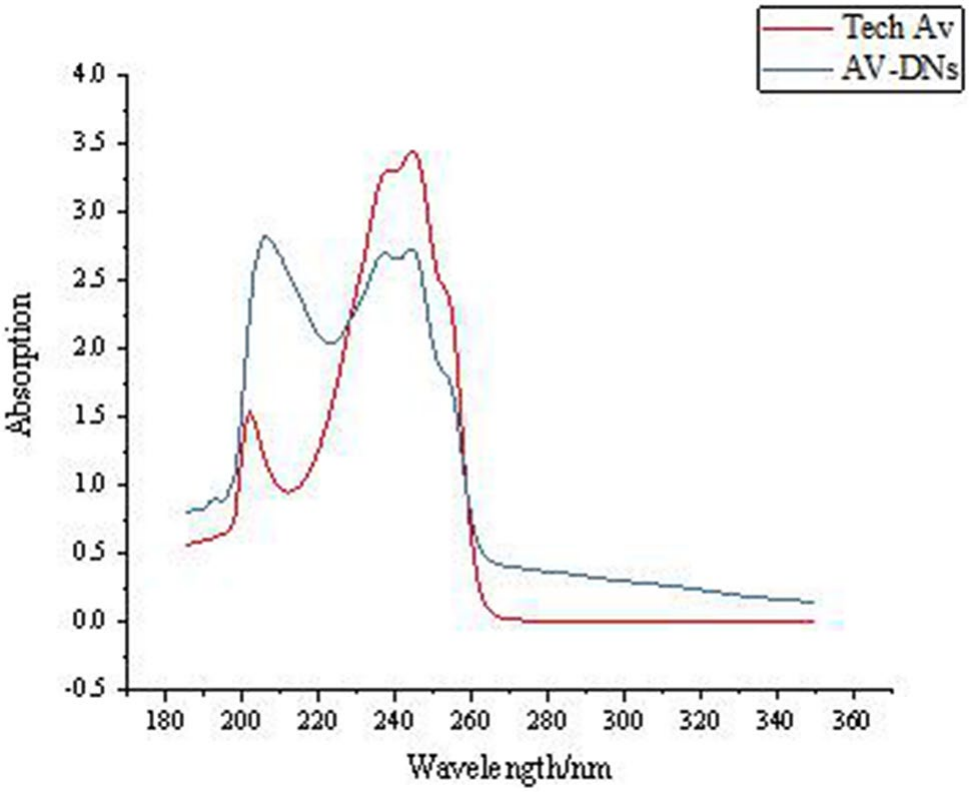
UV–visible spectra of tech. Av and Av-NDs.

The ultraviolet–visible absorption spectrum of Av-NDs and tech. Av is shown in Fig. 5. The UV–visible spectra of tech. Av solution and Av-NDs are completely consistent (Fig. 5). This shows that the Av structure in the nanospheres has not been changed, and the binding between the avermectin and sodium lignosulfonate is only a simple physical entrapment.

**Figure 6:**
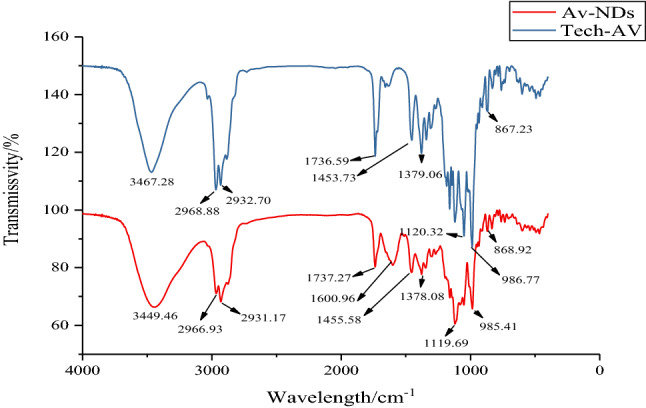
FTIR spectra of Av-NDs and tech. Av.

It can be seen from the infrared spectrum (Fig. 6) of tech. Av and Av-NDs that the carbonyl stretching vibration peak of tech. Av at 1737 cm^−1^ remains unchanged in Av-NDs. The absorption peak appeared at 3467 cm^−1^ in the tech. Av is regarded as the stretching vibration absorption of the hydroxyl group. The same absorption appears at 3449 cm^−1^ in Av-NDs, which is due to the hydroxy stretching vibration absorption also exist in sodium lignosulfonate. Other IR absorption peaks existing in tech. Av are present also in Av-NDs and the positions of the absorption are basically unchanged, indicating that the avermectin molecules have been successfully encapsulated in the carrier. In addition, in Av-NDs, the new absorption peak at 1601 cm^−1^ is the absorption of the N=N double bond in the azobenzene group formed after the crosslinking of sodium lignosulfonate and p-phenylenediamine diazonium salt, which indicates that the cross-linking of sodium lignosulfonate has occurred in Av-NDs.

**Figure 7:**
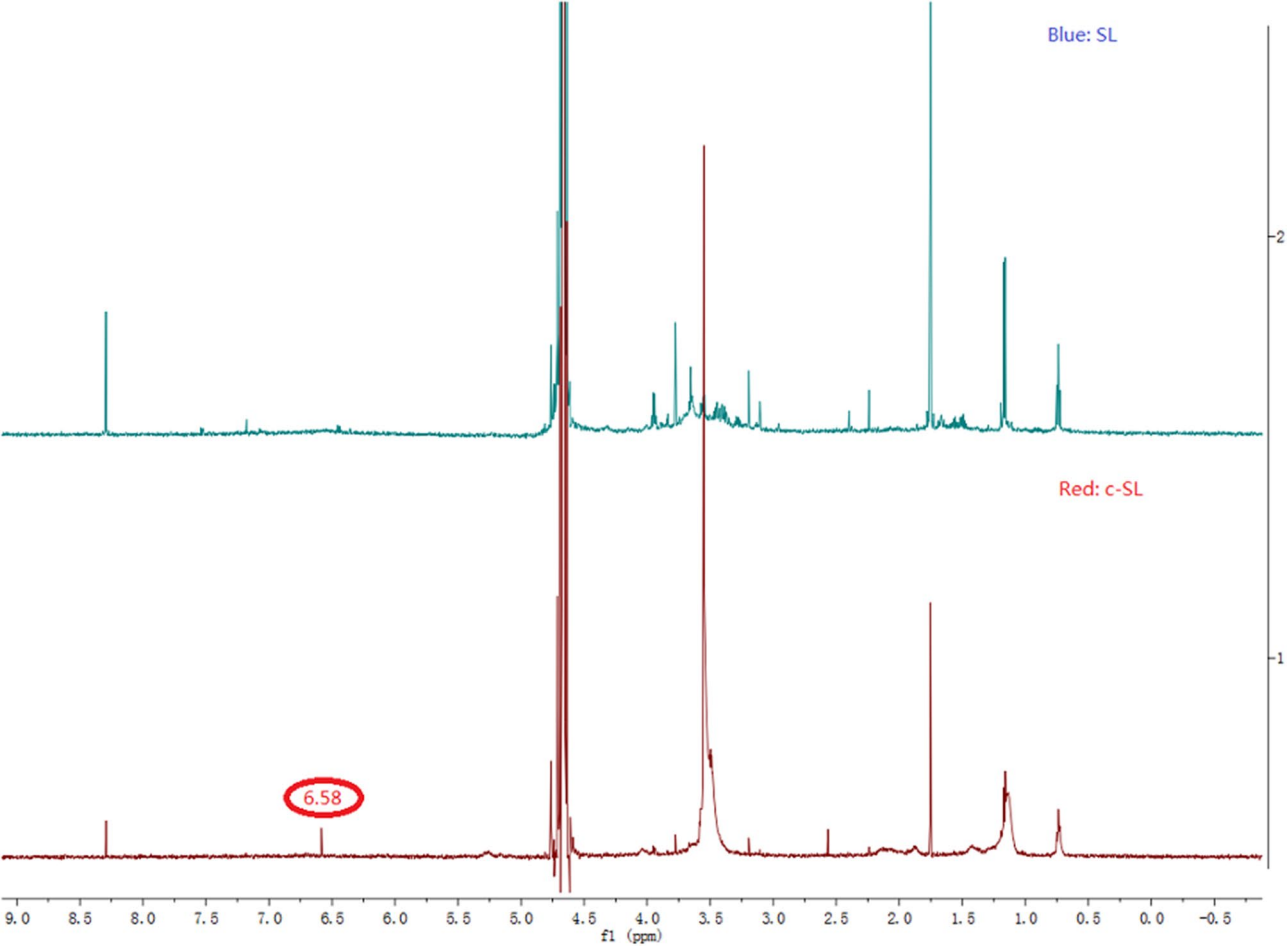
^1^H NMR of the cross-linked sodium lignosulfonate (c-SL) and the sodium lignosulfonate (SL) (The preparation method of c-SL is identical with that of Av-NDs, but avermectin was not added during the preparation process).

The ^1^H NMR spectra of sodium lignosulfonate and its product after cross-linking with p-phenylenediamine diazonium salt are shown in Fig. 7. After cross-linking of sodium lignosulfonate with p-phenylenediamine diazonium salt, a relatively strong absorption peak appeared in the low field with a chemical shift of 6.58 ppm (solvent: D_2_O). This is the chemical shift of hydrogen on the benzene ring after the cross-linking of the p-phenylenediamine diazonium salt with the sodium lignosulfonate because the chemical shift of hydrogen on the benzene ring of the p-phenylenediamine is 6.63 ppm, while the chemical shift of hydrogen on the benzene ring of the hydroquinone from the hydrolysis of diazonium salt is 6.67 ppm, proving that hydrolysis of diazonium salt did not took place and the cross-linking reaction between the p-phenylenediamine diazonium salt and the sodium lignosulfonate indeed occurred.

### Storage stability

The storage stability of Av-NDs was determined by comparison the size of nanoparticles, PDI and AV content before and after 14 days storing at room temperature, 4 °C and 54 °C. As shown in Table 1, the particle size of Av-NDs increased by 8.1 nm, 7.0 nm, and 12.0 nm, respectively and the PDI almost remained unchanged. Besides, the Av content nearly remained unchanged as well, which was 3.805%, 3.801% and 3.857% after storage for 14 days at room temperature, 4 °C and 54 °C, respectively. These results illustrated that Av-NDs has excellent storage stability.

**Table 1:** Storage stability test of the Av-NDs.

Sample	Storage conditions	Particle size /nm	PDI	Content of AV (%)
Av-NDs	Before storage	121.4 ± 1.1	0.244 ± 0.014	3.813
	Room temp	129.5 ± 2.8	0.254 ± 0.031	3.805
	4 °C	128.1 ± 0.5	0.251 ± 0.019	3.801
	54 °C	133.4 ± 1.2	0.311 ± 0.015	3.817

### Release behavior of Av for Av-NDs

**Figure 8:**
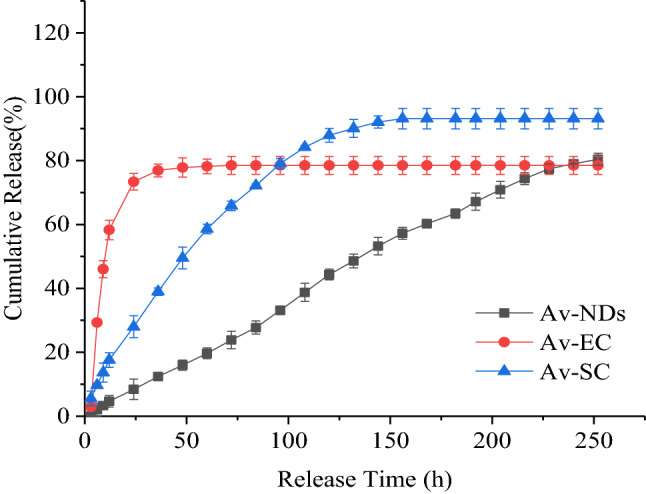
Release curve of Av-NDs in 70% methanol aqueous solution.

Controlled release of active ingredients in pesticide formulations is an important indicator of intelligent pesticide formulations. In order to explore the Av release behaviors of Av-NDs, a systematic investigation was conducted for Av-NDs under different pH conditions, and the commercial Av-EC and Av-SC were used as controls. Because the solubility of avermectin is very poor in water, methanol–water mixed solvent (CH_3_OH:H_2_O/7:3, v/v) is used to increase its dissolution capacity. The result showed that Av-NDs have a longer release time than both Av-EC and Av-SC (Fig. 8). After the first 9 h of release, the release rate of Av-NDs was 3.29%, and the release rates of Av-EC and Av-SC were 46.01% and 13.64%, respectively. It demonstrates that at the early stage of release, Av could not be easily released in Av-NDs because it is embedded in crosslinked sodium lignosulfonate and that the free Av was released predominantly.

After 24 h of release, the release rate of Av-NDs was 8.39%, while the release rates of Av-EC and Av-SC were 73.39% and 28.00%, respectively. The release rate of Av-EC and Av-SC is 8.74 and 3.34 times of Av-NDs. Furthermore, Av-EC almost released completely during 48 h and Av-SC released completely during 120 h since no Av could be detected in the further samples. The reason may be that during the test, Av is easily decomposed in Av-EC and Av-SC, resulting in a deviation of the measured value and a 100% cumulative release rate is not detected.

However, the release rate of Av in Av-NDs was 44.33% at 120 h, and was 70.88% and 79.33% at 204 h and 240 h, respectively, indicating that Av continued to be released. In other words, after the cross-linking of sodium lignosulfonate with p-phenylenediamine diazonium salt, Av molecules were embedded in the nanoparticles formed, and the Av in Av-NDs could be smoothly controlled to release. At the same time, due to the presence of sodium lignosulfonate, the stability of Av was greatly improved.

**Figure 9:**
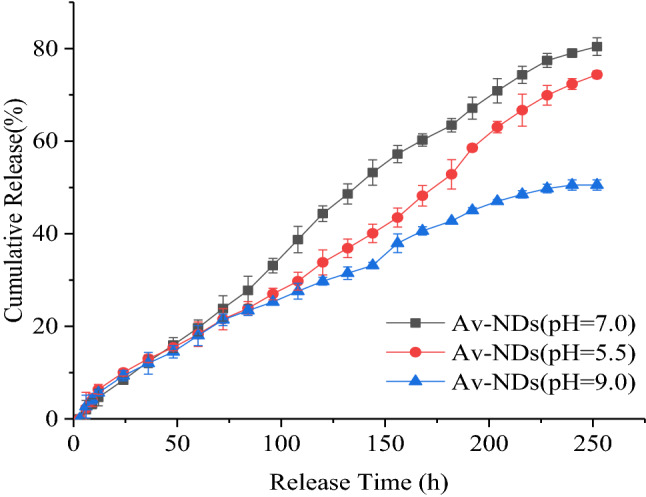
Release curve of Av-NDs in 70% methanol with different pH.

Figure 9 is the release curve of Av-NDs in 70% methanol aqueous solution under different pH conditions. It can be seen from Fig. 9 that for Av-NDs, the release of Av has a characteristic of pH-responsive, and the release rate of Av is: neutral conditions > acidic conditions > alkaline conditions. For example, at the release time of 204 h, the release rate of Av was 70.88% under neutral conditions, and 63.05% and 47.02% for acidic and alkaline, respectively. The release rate of Av under neutral condition is higher than that under acidic condition, and the release rate under acidic conditions is higher than that under alkaline condition, showing that the release of Av in Av-NDs has a pH response and the release rate of Av can be controlled by adjusting the pH value of the medium. The reason and release mechanism of this pH-responsive controlled-release need to be further studied.

In order to understand the release mechanism of Av in Av-NDs, the Ritger-Peppas equation was used to fit the release profile: *M*_*t*_/*M*_∞_ = *kt*^*n*^. Where *Mt* is the Av cumulative release at time *t*, *M*_∞_ is the cumulative release at an infinite time, *k* is the release rate constant, and *n* is the release exponent. A value of *n* < 0.43 is characteristic of Fickian diffusion, *n* > 0.85 corresponds to case II diffusion, and 0.43 < n < 0.85 is indicative of non-Fickian release^36^.

The release kinetic constant *k*, diffusion index *n* and correlation coefficient obtained by fitting the experimental data with the Ritger-Peppas equation are shown in Table 2.

**Table 2:** Ritger-Peppas fitting results of Av-NDs release in water.

Sample condition	Fitting equation	R^2^	*K*	*n*
pH = 5.5	M_t_/M_∞_ = 0.0029t^0.18^	0.9932	0.0029	1.00
pH = 7.0	M_t_/M_∞_ = 0.0056t^0.94^	0.9911	0.0056	0.90
pH = 9.0	M_t_/M_∞_ = 0.0090t^0.45^	0.9921	0.0090	0.73

It can be seen from the table that the release index n of Av at pH = 5.5 and pH = 7.0 is 1.00 and 0.90, respectively, indicating that the release mechanism of Av-NDs is mainly case II diffusion and Av is mainly released by matrix corrosion. For the alkaline condition of pH = 9.0, the release index n is 0.73, indicating that the release mechanism of Av-NDs is mainly non-Fick diffusion and diffusion and matrix erosion coexist during releasing.

### UV-shielding properties of Av in Av-NDs

**Figure 10:**
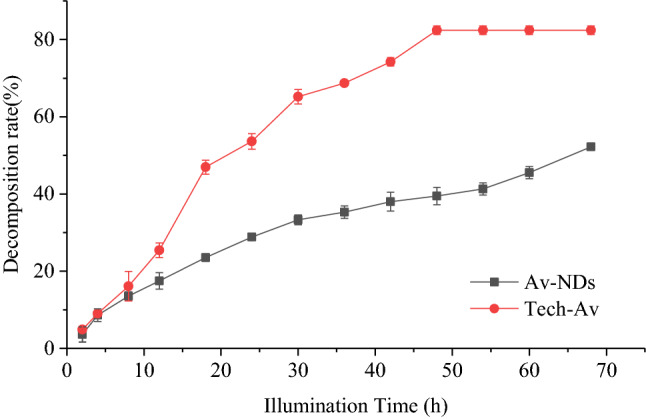
Comparison of the UV-Shielding Properties of Av-NDs with tech. Av.

To determine the UV-shielding properties of Av in Av-NDs, the photolytic rate of Av was assessed by artificial irradiation (Fig. 10). The irradiation time for Av decomposition to 50% in tech. Av was 24 h, while for the Av in Av-NDs was extended to 68 h. The decomposition rate of Av in Av-NDs is 2.8 times slower than that in tech. Av. When exposed to UV light for 48 h, the photolysis percentage of Av in tech. Av was 82.43%, and the photolysis percentage of Av-NDs was 39.48%. These results indicate that Av-NDs shows better anti-photolysis performance than tech. Av. This is because sodium lignosulfonate, a carrier of the Av, has good antioxidant property and exerts a good anti-photolysis effect on Av-NDs. Since Av-NDs has better stability against UV light decomposition, which is helpful to reduce the decomposition loss of Av in field application, and therefore improve the utilization efficiency of the formulation.

### Anti-pest activity assay

**Figure 11:**
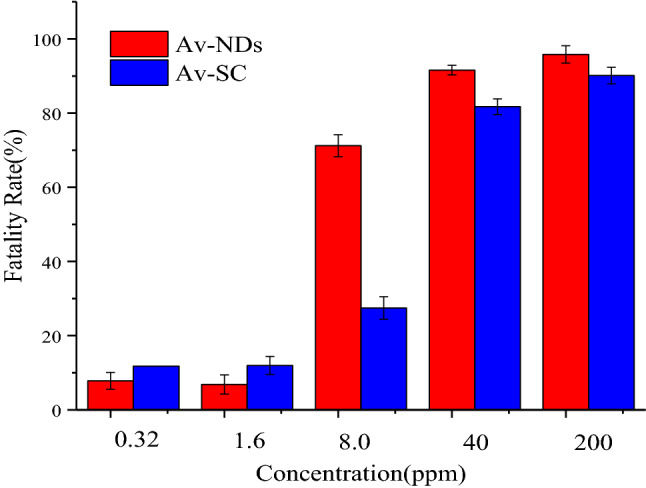
Comparison of toxicity of Av-NDs and Av-SC to *Mythimna separata* at different concentrations.

In this work, the toxicity of Av-NDs were evaluated using *Mythimna separate* as a modal pest, maize leaves as the food for *Mythimna separate*, and commercial Av-SC as the controls. Figure 11 displayed that when the Av concentration is 200 ppm and 40 ppm, the insecticidal effect of Av-NDs on *Mythimna separata* is nearly identical to that of Av-SC, which is close to 100%. When the Av concentration was reduced to 8 ppm, the insecticidal activity of Av-NDs was higher than that of Av-SC. At the time, the insecticidal efficiency of Av-NDs was 71.20%, significantly higher than the 27.45% of Av-SC. The result shows that the Av in Av-NDs has stronger insecticidal activity and is more stable than the Av in Av-SC.

## Conclusions

Using high-speed emulsification and ultrasonic dispersion technology, and embedding the pesticide molecule through the cross-linking reaction between sodium lignosulfonate and p-phenylenediamine diazonium salt, the avermectin nano-delivery system (Av-NDs) with a particle size of 80–150 nm was prepared. Optimization of the influence of the type and amount of emulsifiers and dispersants on the particle size and stability of the nanoformulation showed that when By-125 was used as the emulsifier, 500-LQ as the dispersant and sec-butyl acetate as the solvent of Av, Av-NDs had a small particle size and good stability. The results of the anti-photolysis and controlled-release tests indicate that Av-NDs shows better anti-photolysis performance and controlled-release ability than Av-SC, and Av-NDs is functionalized with pH-responsive controlled release. The release rate in different pH media is neutral > acidic > alkaline, and the release rate in neutral media is twice that in alkaline media. The result of the insecticidal activity test shows that Av-NDs possesses better insecticidal effect than Av-SC. The Av-NDs with anti-photolysis and controlled-release characteristics is suitable for large-scale industrial production and is capable to be utilized as effective insecticide in the field.

## Supplementary Information

Supplementary Information.
