# Supplementary material for: Fabrication and evaluation of slow-release lignin-based avermectin nano-delivery system with UV-shielding property
Source: Sci Rep. 2021 Dec 1;11:23248. doi: 10.1038/s41598-021-02664-7 (PMC8636473; doi:10.1038/s41598-021-02664-7)
Supplement: Supplementary file 1 — Supplementary Information. [file 41598_2021_2664_MOESM1_ESM.docx]

**SUPPLEMENTARY MATERIALS**

**Fabrication and Evaluation of slow-release Lignin-Based Avermectin Nano-Delivery System with** **UV-Shielding Property**

Dongmei Mo^1#^ Xiangying Li^1#^ Yong Chen^1^ Yang Jiang^2^ Chunfang Gan^1^ Yuanfei Zhang^1^ Weiguo Li^2^ Yanmin Huang^1*^ Jianguo Cui^1,2*^

^1^ Guangxi Key Laboratory of Natural Polymer Chemistry and Physics, Nanning Normal University, Nanning, 530001, PR China;

^2^ Guangxi Tianyuan Biochemical Co. Ltd., Nanning, 530001, PR China;

^#^ DM. Mo and XY. Li contributed equally to this work.

*Corresponding author at: College of Chemistry and Material Science, Nanning Normal University, Nanning 530001, China. E-mail address: [cuijg1954@126.com](mailto:cuijg1954@126.com) (JG. Cui); huangyanmin828@163.com (YM. Huang).

**ABSTRACT**

Nanopesticide is one of the best pesticide formulation technologies to overcome the disadvantages of traditional pesticides, which has received great attention from the international community．Using high-speed emulsification and ultrasonic dispersion technology, an avermectin nano-delivery system (Av-NDs) with a particle size of 80-150nm was prepared through embedding the pesticide molecule utilizing the cross-linking reaction between sodium lignosulfonate and p-phenylenediamine diazonium salt. The formulation and composition of Av-NDs were optimized, the morphology of Av-NDs was analyzed by scanning electron microscope (SEM), transmission electron microscope (TEM) and dynamic light scattering (DLS), and the structure of Av-NDs was characterized by UV, IR and ^1^H NMR. Anti-photolysis and controlled-release tests show that the stability of Av-NDs is 3-4 times of the original avermectin (Av) and possesses the pH-responsive controlled release property. Furthermore, the insecticidal activity of Av-NDs is better than that of avermectin suspension concentrate (Av-SC). The Av-NDs with anti-photolysis and controlled-release characteristics is suitable for large-scale industrial production and is capable to be utilized as effective insecticide in the field.

**KEYWORDS**

Avermectin; Sodium lignosulfonate; Nanopesticide; Controlled-release; UV-Shielding.

**Supporting Information**

**Contents**

1. Figure S1 Particle size distribution diagram of the Av-NDs S2
2. Figure S2 Storage stability change of the Av-NDs S2
3. Figure S3 ^1^H NMR of p-Phenylenediamine and Hydroquinone S3
4. Table S1 The influence of solvent type on particle size S4
5. Table S2 The influence of different emulsifiers on the Av-NDs S4
6. Table S3 The influence of different dispersants on the Av-NDs S5
7. Table S4 The cumulative release of the Av-NDs in 70% methanol aqueous solution S6
8. Table S5 The cumulative release of the Av-NDs in 70% methanol with different pH S7
9. Table S6 Comparison of the UV-Shielding Properties of the Av-NDs with tech. Av S8
10. Table S7 Comparison of toxicity of the Av-NDs and Av-SC to *Mythimna separata* at different concentrations S8

S1


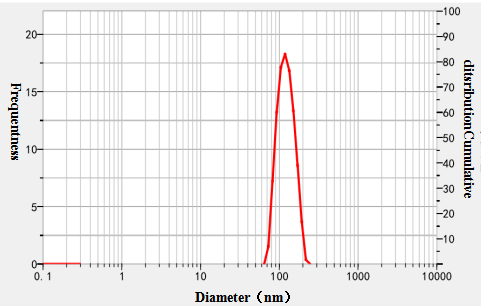


Figure S1 Particle size distribution diagram of the Av-NDs


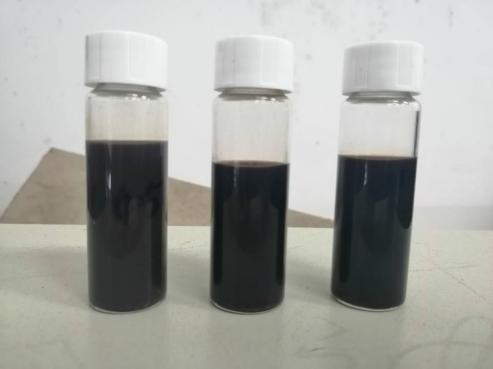

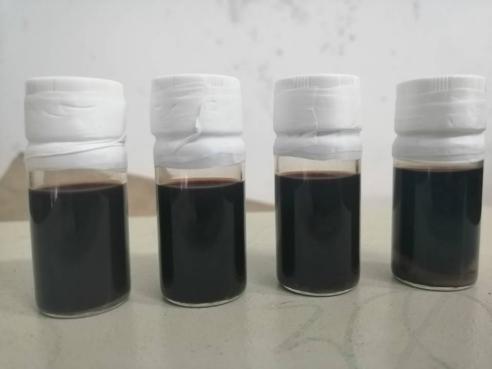


After the cold storage After the hot storage

Figure S2 Storage stability change of the Av-NDs

S2

Figure S3 ^1^H NMR of p-Phenylenediamine and Hydroquinone

^1^H NMR of p-Phenylenediamine (Solvent: D_2_O):

^1^H NMR of Hydroquinone (Solvent: D_2_O):

S3

Table S1 The influence of solvent type on particle size*

| Solvents | Particle size /nm | PDI | Stability* |
| --- | --- | --- | --- |
| s-butyl acetate | 125.9±2.8 | 0.254±0.031 | A |
| methanol | >1000 | - | - |
| cyclohexanone | >1000 | - | - |
| dichloromethane | >1000 | - | - |
| Dupont DBE:N-Ndimethylacetamide=3:1 | >1000 | - | - |
| s-butyl acetate:N-Ndimethylacetamide =2:1 | 113.4±0.5 | 0.219±0.018 | B |
| s-butyl acetate:N-Ndimethylacetamide =1:1 | >1000 | - | - |

*A. The appearance is stable without any change; B. A small amount of aggregates appeared.

C. Emulsifier: By-125; Dispersant: NS-500LQ.

Table S2 The influence of different emulsifiers on the Av-NDs*

| emulsifiers | Particle size /nm | PDI |
| --- | --- | --- |
| Span-80 | >1000 | - |
| Tween -60 | >1000 | - |
| Tween -80 | >1000 | - |
| By-125 | 125.9+2.8 | 0.254+0.031 |

* Solvent: sec-butyl acetate; Dispersant: NS-500LQ.

S4

Table S3 The influence of different dispersants on the Av-NDs*

| Dispersants | Particle size /nm | PDI |
| --- | --- | --- |
| Sodium alginate | 113.3±2.9 | 0.203+0.07 |
| Xanthan gum | 122.9±1.9 | 0.297+0.026 |
| Hydroxypropyl starch ether | >1000 | - |
| Acetylated distarch phosphate | 154.3±12.0 | 0.314+0.032 |
| Hydroxypropyl distarch phosphate | >1000 | - |
| Acetylated distarch adipate | 188.4±1.4 | 0.147+0.01 |
| Distarch phosphate | 133±1.4 | 0.306+0.031 |
| Oxidized starch | 127±2.5 | 0.200+0.068 |
| Sodium hydroxyethyl cellulose | 134.6±0.4 | 0.223+0.021 |
| Guar gum | 134±6.3 | 0.225+0.113 |
| NS-500LQ | 125.9±2.8 | 0.254+0.031 |
| J-601 | >1000 | - |
| SP-SC29 | >1000 | - |
| Darren DSV | >1000 | - |

* Solvent: sec-butyl acetate; Emulsifier: NS-500LQ.

S5

Table S4 The cumulative release of the Av-NDs in 70% methanol aqueous solution (Figure 8)

| Time (h) | Cumulative Release (%) | | |
| --- | --- | --- | --- |
|  | Av-NDs | Av-EC | Av-SC |
| 3 | 0.00±0.000 | 2.91±1.052 | 5.55±2.310 |
| 6 | 1.98±2.004 | 29.30±0.536 | 9.67±0.709 |
| 9 | 3.29±1.087 | 46.01±2.684 | 13.64±2.981 |
| 12 | 4.63±1.812 | 58.29±3.031 | 17.57±2.300 |
| 24 | 8.39±3.297 | 73.39±2.618 | 28.00±3.455 |
| 36 | 12.39±1.311 | 76.93±2.016 | 38.99±1.185 |
| 48 | 15.97±1.582 | 77.84±3.011 | 49.51±5.432 |
| 60 | 19.66±1.710 | 78.19±2.288 | 58.60±1.482 |
| 72 | 23.85±2.723 | 78.49±2.830 | 65.87±1.502 |
| 84 | 27.75±2.071 | 78.49±2.830 | 72.16±0.580 |
| 96 | 33.13±1.306 | 78.49±2.830 | 79.08±1.598 |
| 108 | 38.74±2.846 | 78.49±2.830 | 84.21±0.608 |
| 120 | 44.33±1.695 | 78.49±2.830 | 87.92±2.161 |
| 132 | 48.60±2.179 | 78.49±2.830 | 90.08±2.830 |
| 144 | 53.23±2.726 | 78.49±2.830 | 92.07±1.904 |
| 156 | 57.22±1.849 | 78.49±2.830 | 93.14±3.200 |
| 168 | 60.26±1.325 | 78.49±2.830 | 93.14±3.200 |
| 182 | 63.44±1.449 | 78.49±2.830 | 93.14±3.200 |
| 192 | 67.13±2.671 | 78.49±2.830 | 93.14±3.200 |
| 204 | 70.88±2.622 | 78.49±2.830 | 93.14±3.200 |
| 216 | 74.34±1.851 | 78.49±2.830 | 93.14±3.200 |
| 228 | 77.42±1.515 | 78.49±2.830 | 93.14±3.200 |
| 240 | 79.03±0.100 | 78.49±2.830 | 93.14±3.200 |
| 252 | 80.42±1.900 | 78.49±2.830 | 93.14±3.200 |

S6

Table S5 The cumulative release of the Av-NDs in 70% methanol with different pH (Figure 9)

| Time (h) | Cumulative Release (%) | | |
| --- | --- | --- | --- |
|  | Av-NDs(pH=7.0) | Av-NDs(pH=5.5) | Av-NDs(pH=9.0) |
| 3 | 0.00±0.000 | 0.00±0.000 | 0.00±0.000 |
| 6 | 1.98±2.004 | 2.28±3.488 | 2.66±2.449 |
| 9 | 3.29±1.087 | 4.06±1.386 | 4.18±0.981 |
| 12 | 4.63±1.812 | 6.37±1.074 | 5.65±0.616 |
| 24 | 8.39±0.750 | 10.03±0.766 | 9.33±1.098 |
| 36 | 12.39±1.311 | 13.00±0.809 | 12.02±2.330 |
| 48 | 15.97±1.582 | 15.43±0.687 | 14.49±1.330 |
| 60 | 19.66±1.710 | 18.29±2.662 | 17.99±2.150 |
| 72 | 23.85±2.723 | 21.55±2.295 | 21.41±1.241 |
| 84 | 27.75±3.071 | 23.89±1.455 | 23.42±1.068 |
| 96 | 33.13±1.563 | 26.98±1.242 | 25.26±0.110 |
| 108 | 38.74±2.846 | 29.74±1.932 | 27.59±1.714 |
| 120 | 44.33±1.695 | 33.81±2.685 | 29.74±0.841 |
| 132 | 48.60±2.179 | 36.87±1.997 | 31.48±1.345 |
| 144 | 53.23±2.726 | 40.08±1.989 | 33.15±0.652 |
| 156 | 57.22±1.849 | 43.51±2.044 | 37.95±2.002 |
| 168 | 60.26±1.325 | 48.21±2.212 | 40.63±0.909 |
| 182 | 63.44±1.449 | 52.83±3.180 | 42.75±0.242 |
| 192 | 67.13±2.374 | 58.55±0.708 | 45.06±0.555 |
| 204 | 70.88±2.622 | 63.05±1.247 | 47.02±0.382 |
| 216 | 74.34±1.851 | 66.70±3.445 | 48.52±0.784 |
| 228 | 77.42±1.515 | 69.91±2.149 | 49.76±0.942 |
| 240 | 79.03±0.905 | 72.36±1.169 | 50.53±1.127 |
| 252 | 80.42±1.900 | 74.37±0.798 | 50.53±1.127 |

S7

Table S6 Comparison of the UV-Shielding Properties of the Av-NDs with tech. Av (Figure 10)

| Time (h) | Decomposition Rate (%) | |
| --- | --- | --- |
|  | Av-NDs | Tech-Av |
| 2 | 3.63±1.987 | 4.90±0.216 |
| 4 | 8.61±1.621 | 9.04±0.660 |
| 8 | 13.55±1.131 | 16.09±3.822 |
| 12 | 17.45±2.136 | 25.43±1.893 |
| 18 | 23.51±0.942 | 46.94±1.803 |
| 24 | 28.82±0.624 | 53.62±2.010 |
| 30 | 33.30±1.269 | 65.19±1.891 |
| 36 | 35.28±1.633 | 68.71±0.620 |
| 42 | 37.99±2.449 | 74.22±1.087 |
| 48 | 39.48±2.246 | 82.43±1.087 |
| 54 | 41.30±1.563 | 82.43±1.087 |
| 60 | 45.54±1.584 | 82.43±1.087 |
| 68 | 52.21±0.000 | 82.43±1.087 |
| 74 | 52.21±0.000 | 82.43±1.087 |

Table S7 Comparison of toxicity of the Av-NDs and Av-SC to *Mythimna separata* at different concentrations (Figure 11)

| Concentration  (ppm) | Fatality Rate (%) | |
| --- | --- | --- |
|  | Av-NDs | Av-SC |
| 0.32 | 7.81±2.257 | 11.8±0.000 |
| 1.6 | 6.84±2.564 | 11.97±2.425 |
| 8 | 71.2±2.949 | 27.45±3.022 |
| 40 | 91.58±1.299 | 81.73±2.114 |
| 200 | 95.83±2.357 | 90.12±2.254 |

S8
